# Supplementary material for: Multi‐dataset identification of innovative feature genes and molecular mechanisms in keratoconus
Source: J Cell Mol Med. 2024 Sep 19;28(18):e70079. doi: 10.1111/jcmm.70079 (PMC11412914; doi:10.1111/jcmm.70079)
Supplement: Supplementary file 2 — Material S2. [file JCMM-28-e70079-s002.zip › 7.GSEA analysis/1.ARL11/KEGG_HOMOLOGOUS_RECOMBINATION.html]

Details for gene set KEGG\_HOMOLOGOUS\_RECOMBINATION[GSEA]

|  || Dataset | ARL11.group.cls#h\_versus\_l |
| Phenotype | group.cls#h\_versus\_l |
| Upregulated in class | h |
| GeneSet | KEGG\_HOMOLOGOUS\_RECOMBINATION |
| Enrichment Score (ES) | 0.29659057 |
| Normalized Enrichment Score (NES) | 0.98158073 |
| Nominal p-value | 0.44705883 |
| FDR q-value | 1.0 |
| FWER p-Value | 1.0 |
Table: GSEA Results Summary

  

Fig 1: Enrichment plot: KEGG\_HOMOLOGOUS\_RECOMBINATION      
 Profile of the Running ES Score & Positions of GeneSet Members on the Rank Ordered List

  

| SYMBOL | TITLE | RANK IN GENE LIST | RANK METRIC SCORE | RUNNING ES | CORE ENRICHMENT || 1 | XRCC2 | na | 1475 | 0.339 | 0.1395 | Yes |
| 2 | RPA4 | na | 5183 | 0.160 | 0.1187 | Yes |
| 3 | BRCA2 | na | 5798 | 0.142 | 0.1774 | Yes |
| 4 | TOP3B | na | 7555 | 0.101 | 0.1808 | Yes |
| 5 | RAD54L | na | 8564 | 0.082 | 0.1956 | Yes |
| 6 | XRCC3 | na | 8577 | 0.081 | 0.2389 | Yes |
| 7 | BLM | na | 8980 | 0.075 | 0.2677 | Yes |
| 8 | RAD54B | na | 10083 | 0.059 | 0.2676 | Yes |
| 9 | RPA1 | na | 10886 | 0.049 | 0.2707 | Yes |
| 10 | TOP3A | na | 10899 | 0.049 | 0.2964 | Yes |
| 11 | POLD1 | na | 11636 | 0.040 | 0.2966 | Yes |
| 12 | SSBP1 | na | 14376 | 0.016 | 0.2262 | No |
| 13 | RAD51C | na | 15261 | 0.009 | 0.2057 | No |
| 14 | RAD52 | na | 15571 | 0.007 | 0.2006 | No |
| 15 | POLD4 | na | 16051 | 0.003 | 0.1886 | No |
| 16 | RAD51D | na | 17031 | -0.003 | 0.1622 | No |
| 17 | MUS81 | na | 17062 | -0.004 | 0.1633 | No |
| 18 | RAD50 | na | 17438 | -0.006 | 0.1558 | No |
| 19 | RPA2 | na | 18069 | -0.011 | 0.1435 | No |
| 20 | POLD3 | na | 18840 | -0.016 | 0.1298 | No |
| 21 | NBN | na | 23757 | -0.059 | 0.0200 | No |
| 22 | RPA3 | na | 23802 | -0.060 | 0.0508 | No |
| 23 | POLD2 | na | 24003 | -0.062 | 0.0784 | No |
| 24 | RAD51 | na | 27599 | -0.121 | 0.0398 | No |
| 25 | RAD51B | na | 28884 | -0.154 | 0.0854 | No |
| 26 | EME1 | na | 28923 | -0.155 | 0.1673 | No |
Table: GSEA details [plain text format]

  

Fig 2: KEGG\_HOMOLOGOUS\_RECOMBINATION      
 Blue-Pink O' Gram in the Space of the Analyzed GeneSet

  

Fig 3: KEGG\_HOMOLOGOUS\_RECOMBINATION: Random ES distribution      
 Gene set null distribution of ES for **KEGG\_HOMOLOGOUS\_RECOMBINATION**
